# The Impact of Accented Input on Spanish-English Bilingual Children’s Word Learning

**DOI:** 10.3390/bs16060943

**Published:** 2026-06-09

**Authors:** Milijana Buac, Margarita Kaushanskaya

**Affiliations:** 1 School of Allied Health and Communicative Disorders, Northern Illinois University, DeKalb, IL 60115, USA; 2 Department of Communication Sciences and Disorders, University of Wisconsin-Madison, Madison, WI 53706, USA; kaushanskaya@wisc.edu

**Keywords:** bilingual children, accented input, word-learning

## Abstract

Background: Bilingual children are frequently exposed to accented speech, yet it remains unclear how accent familiarity affects their ability to learn new words. This study examined Spanish–English bilingual children’s (*n* = 46) novel word learning from speakers with familiar and unfamiliar accents and investigated individual differences related to learning from accented input. Methods: Children completed an experimental word-learning task in which they learned novel word–object pairings produced by three speakers: a speaker of General American English, a Spanish-accented English speaker (familiar accent), and a Korean-accented English speaker (unfamiliar accent). Individual-differences analyses examined associations between learning outcomes and children’s language skills, length of bilingualism, and characteristics of language input in the home environment. Results: Children showed more difficulty learning novel words from the unfamiliar Korean-accented speaker than from the familiar Spanish-accented speaker and the General American-English speaker. Language skills were associated with learning from the familiar accent but not the unfamiliar accent. Length of bilingualism was positively associated with learning from the unfamiliar accent, whereas greater strength of foreign-accented English in the environment was negatively associated with learning from the native speaker. Conclusions: These findings suggest that accent familiarity facilitates bilingual children’s word learning and that experience-related factors contribute to their ability to accommodate accent variability.

## 1. Introduction

The bilingual environment is characterized by immense linguistic variability, and children may be exposed to native and non-native speakers of their languages who vary in their levels of language proficiency (Place & Hoff, 2011; Unsworth, 2016). It is, therefore, common for bilingual children to hear accented^1^ language input. This exposure may have an impact on how children acquire information embedded in accented input. Yet, most previous studies addressing the impact of accented language input have been conducted with monolingual participants, despite the possibility that the bilingual environment may augment processing of accented input (Pinet et al., 2011). Further, previous studies primarily focused on how foreign accents impact language processing, whereas the question of how foreign accents impact the ability to acquire new information has received much less attention. Therefore, the goal of the present study was to examine bilingual Spanish-English children’s ability to learn novel words from accented speakers.

### 1.1. The Impact of Accented Input on Bilinguals’ Processing and Comprehension

Numerous studies have shown that when monolingual speakers are exposed to accented input in laboratory settings, even when this exposure is brief, there are measurable improvements in their ability to recognize words (Potter & Saffran, 2017) and learn words (Schmale et al., 2011) from accented input. Logically, then, lifelong exposure to accented input, as would be the case for many bilinguals, might also result in similar accommodation. Indeed, a few studies that have examined language processing under accented vs. unaccented conditions in bilingual adults. (Bent & Bradlow, 2003; Hanulíková & Weber, 2012; Levy et al., 2019; Pinet et al., 2011) have observed just such a result. These studies also demonstrated that intelligibility of accented speech was higher when the listener’s and the speaker’s language backgrounds matched, perhaps because of shared phonological knowledge (Bent & Bradlow, 2003) or because of the listener’s experience with listening to accented input (Adank et al., 2009). More broadly, accent has been shown to influence not only lexical processing but also higher-level linguistic processes such as syntactic priming (Chun & Kaan, 2022). However, in a study assessing children’s ability to comprehend accented vs. unaccented sentences, McDonald et al. (2018) demonstrated largely similar effects of Spanish-accented English on language processing across three different groups of children characterized by different degrees of experience with Spanish: monolingual English-speaking children, simultaneous Spanish-English bilinguals, and early Spanish-English bilinguals. That is, all three groups of children experienced similar processing costs associated with Spanish-accented English despite the fact that the bilingual groups shared a language background with the accented speaker.

Several other studies have included individuals with extensive experience with foreign accents and assessed their ability to process or comprehend unfamiliar foreign accents (Evans & Tomé Lourido, 2019; Levy et al., 2019; Tao & Taft, 2016). Tao and Taft (2016) showed that young adults who have been exposed to accented input throughout their lives experienced difficulty processing an unfamiliar accent, similar to their peers with no experience with accented input. Similarly, Evans and Tomé Lourido (2019) did not find an advantage in comprehension of unfamiliar accents in five- and six-year-old bilingual children when compared to their monolingual peers. Lastly, Levy et al. (2019) assessed the impact of the type of accent and the amount of experience on school-age bilingual children’s ability to process an unfamiliar foreign accent and an unfamiliar regional dialect. Their findings showed that only experience with regional dialect aided children in the regional dialect condition, such that children with more experience with regional dialect were better able to process sentences in this condition. However, experience with foreign accents did not aid in children’s processing of sentences from an unfamiliar accented speaker. Together, these studies demonstrate that bilingual experience and/or experience with accented input does not necessarily lead to improvements in processing and comprehending accented input, both for familiar accents (McDonald et al., 2018) and unfamiliar accents (Evans & Tomé Lourido, 2019; Levy et al., 2019).

However, while aspects of non-native input, such as foreign accent, have been shown to interfere with language processing, little is known about the impact of accented input on children’s learning, especially in bilingual children. Research with monolingual children has demonstrated that children as young as two years of age are able to learn from accented input under certain conditions (Schmale, Cristia, & Seidl, 2012; Schmale, Hollich, & Seidl, 2011; Newman et al., 2018). These studies showed that two-year-old children were not able to learn novel words when they were taught by a native English speaker and tested by a Spanish-accented English speaker, but they succeeded in a reversed condition (Schmale et al., 2011). Slightly older children, 2;6 year-old toddlers, were successful in learning novel words irrespective of the speaker’s background at teaching vs. testing. In a follow-up study, Schmale et al. (2012) showed that brief exposure to accented input helped two-year-old toddlers to learn new information from accented speakers. The successful ability to learn from accented speakers in these studies may be attributed to the phonology of the novel words, that is, the phonological structure of the words that overlapped between English and Spanish. Indeed, Newman et al. (2018) extended Schmale et al.’s (2011) findings by demonstrating that young monolingual children were able to successfully accommodate to accented input only when the language of the accent closely resembled the phonology of the children’s native language. Because bilingual children have an increased likelihood of receiving linguistic input from non-native speakers, vis-à-vis monolingual children, it is possible that non-native input influences learning differently in bilingual children than in monolingual children.

Bilingual children grow up managing variability not only across speakers and accents but also across languages, phonological systems, and lexical inventories. As a result, bilingualism has been hypothesized to confer advantages in processing variable speech input (Byers-Heinlein & Fennell, 2014; Singh et al., 2018). Research on bilingual infants and children indicates that they are often more flexible in accommodating phonetic and phonological variation, which may support learning in variable linguistic environments. Naturalistic studies further underscore the complexity of accented input effects. Although greater exposure to non-native input has sometimes been associated with weaker language outcomes in bilingual children, these associations are modest, and children’s performance typically remains within the average range (Buac & Kaushanskaya, 2023). Importantly, accented input does not prevent successful word learning but may pose additional processing demands, particularly in contexts requiring rapid or precise lexical encoding (Braun et al., 2021).

### 1.2. Factors Impacting Performance on Tasks Involving Accented Input

Across both experimental and naturalistic studies, individual differences play a critical role in shaping children’s learning from accented input. Besides exposure to accent, several other factors, such as age and vocabulary skills, have been examined in relation to children’s performance on a variety of tasks involving accented input. Studies have shown that older children outperform younger children on tasks involving processing, comprehension, and learning from accented input (Mulak et al., 2017; Nathan et al., 1998; O’Connor & Gibbon, 2011; Schmale et al., 2011; Schmale & Seidl, 2009; Van Heugten et al., 2015). Further, children with better vocabulary skills tend to outperform children with poorer vocabulary skills on tasks involving processing and comprehension of accented input (Banks et al., 2015; Bent, 2014; Bent et al., 2016; Janse & Adank, 2012; McDonald et al., 2018). Together, these findings suggest that performance on tasks involving accented input is shaped by both listener-related factors, such as language skills and experience, and characteristics of the input. However, most prior work has focused on processing and comprehension of accented speech, with relatively little attention to how these factors influence children’s ability to learn new information from accented speech.

### 1.3. The Current Study

Building on prior work demonstrating that accented input can affect both language processing and comprehension, the aim of the present study was to assess how bilingual children learn novel words from accented speakers. The study specifically focused on learning rather than processing because learning is more effortful than processing, and therefore may be especially vulnerable to the effects of a foreign accent. Whereas processing of accented speech primarily involves real-time perceptual adaptation and recognition, word learning requires additional processes, including phonological encoding, the formation of stable lexical representations, and the mapping of these representations onto referents. As a result, learning from accented input may be particularly sensitive to variability in the phonological signal. While bilingual children do not show a benefit for processing unfamiliar accents in previous studies, they may show benefits for learning from accented input.

To assess learning from non-native input, a within-subjects design was implemented where four- and five- year-old Spanish-English bilingual children were asked to learn three sets of novel words paired with novel objects from three different speakers: a General American-English speaker, a Spanish-accented English speaker, and a Korean-accented English speaker. Two different foreign accents were included in order to assess whether bilingualism and/or experience with Spanish-accented input would impact learning only from a familiar accent, Spanish-accented English, or whether it might also impact learning from an unfamiliar accent, Korean-accented English. Given that studies assessing bilingual children’s ability to comprehend and process accented input did not find advantages in either a familiar accent (McDonald et al., 2018) or an unfamiliar accent (Levy et al., 2019), it was hypothesized that children in the present study may experience difficulty learning from both the familiar accent and the unfamiliar accent. However, it was also anticipated that they might benefit from accent familiarity and demonstrate superior learning from the speaker with a familiar accent vs. the unfamiliar accent. Therefore, children were expected to have the most success learning from the General American-English speaker, followed by the Spanish-accented speaker, and children were expected to be least successful in learning from the Korean-accented speaker.

The role of individual differences was also examined with a focus on children’s language skills and key aspects of the bilingual experience. Language skills were expected to contribute to children’s ability to learn from both the familiar and unfamiliar accented speakers, in line with prior research on accented speech processing. Previous work has demonstrated that bilingual experience does not uniformly support processing of unfamiliar accents (Levy et al., 2019), but given the heterogeneity of bilingual development (Unsworth, 2016), it is possible that specific dimensions of bilingual experience may influence learning. Accordingly, three bilingual variables were assessed: children’s length of bilingualism, the number of non-native English speakers in the child’s environment (i.e., Spanish-accented English), and the strength of foreign-accented English (i.e., Spanish-accented English) in the child’s environment. These variables were selected based on prior findings linking length of bilingualism to cognitive outcomes (Kapa & Colombo, 2013; Luk et al., 2011) and exposure to non-native input to language outcomes (Place & Hoff, 2011, 2016) and were intended to capture variability in children’s experience with Spanish-accented input. They index broader bilingual experience and exposure to accented input, and are conceptually distinct from familiarity with a specific accented speaker. In particular, accent familiarity may be expected to facilitate learning from a given speaker, whereas broader bilingual experience may support more general flexibility in accommodating phonological variability. Thus, it was hypothesized that the bilingual variables would be associated with learning performance in the familiar Spanish-accented condition, but may also be linked with learning in the unfamiliar Korean-accented condition.

## 2. Materials and Methods

### 2.1. Participants

A total of 57 Spanish-English bilingual children were recruited for the present study. Of the 57 children tested, 11 were excluded from the study, resulting in a total of 46 participants between the ages of 4 and 5 years old (*M* = 4.88, *SD* = 0.60; *Range*: 4.00–5.83; 17 boys and 29 girls). Three children were excluded because they did not complete all three visits required for the present study; one child was excluded due to extensive exposure to a third language early in life, two children were excluded due to malfunction of the experimental equipment resulting in extensive data loss, and five children were excluded due to non-compliance with task instructions and excessive movement resulting in extensive data loss. Children’s language acquisition history, educational background, and medical background were obtained via a parent interview. Per parent report, all children were typically developing. Socioeconomic status was indexed by averaging the total number of years of education of the child’s parents. In the event that the child had only one primary caregiver, that primary caregiver’s total years of education was taken to index socioeconomic status. Parent education ranged widely, but most children had parents with some post-secondary education (*M* = 14.42, *SD* = 4.83, *Range*: 4–24 years). All children passed a bilateral hearing screening at 20 dB at 1000 Hz, 2000 Hz, and 4000 Hz using an Earscan 3 audiometer (Micro Audiometrics Corporation, Daytona Beach, FL, USA).

Of the 46 participants, 43 were identified as Hispanic, while 3 were identified as non-Hispanic. The racial breakdown of the group was as follows: 26 children were identified as White, one child was identified as having more than one race, two children’s parents did not wish to disclose their child’s race, and 17 children’s parents listed Hispanic again as the child’s race. Most of the children were born in the U.S.; specifically, 37 children were born in the U.S., one child was born in Venezuela, one child was born in Costa Rica, one child was born in Honduras, one child was born in Mexico, one child was born in Spain, two children were born in Puerto Rico, and two children were born in Chile. On average, children began acquiring English around 9 months (*M* = 9.74 months, *SD* = 13.22) and Spanish from birth (*M* = 1.22 months, *SD* = 7.10). All children were exposed to English no later than 36 months of age, and could thus be designated as simultaneous bilinguals. For 11 of the children, parents listed English as the child’s first language, for 30 of the children, Spanish was listed as the first language, and for five of the children, parents listed both English and Spanish as the child’s first languages.

Parents were also asked to indicate which language was the child’s dominant language. For 20 of the children, English was listed as the child’s dominant language, for another 20 children, Spanish was listed as the dominant language, and for six of the children, both English and Spanish were listed as dominant languages. In the home environment, 10 children spoke mostly English, 20 children spoke mostly Spanish, and 16 children spoke both English and Spanish. When parents were asked to list the language(s) their child heard at home, four parents listed English as the language their child mainly heard at home, 31 listed Spanish, and 11 listed both English and Spanish. Per parent report, most children had lifelong exposure to speakers of Spanish-accented English. Exposure to accented input was measured using a 5-point scale with 1 being “no exposure or only brief casual exposure” and 5 being “daily at home exposure” (*M* = 3.9, *SD* = 1.32). Seven children were listed as having brief exposure, seven children were listed as having moderate exposure, nine children were listed as having frequent exposure, and 23 children were listed as having daily at-home exposure. With regard to exposure to Korean-accented English speakers, only two children had a brief encounter with a Korean-accented speaker. These children’s data were retained because the exposure was very brief, and the children did not interact directly with the speakers. Numerically, these children’s data (i.e., proportion of looks to the target) did not differ from the rest of the group.

### 2.2. Materials

#### 2.2.1. Standardized Measures

To assess children’s language skills, the Preschool Language Scale, Fifth Edition Spanish (PLS-5, Zimmerman et al., 2012) was used. This assessment provides conceptual scores where children were first assessed in Spanish, and then any item that was responded to incorrectly in Spanish was re-administered in English. Thus, children’s total language knowledge across both languages was assessed. The PLS-5 Spanish provides three standard scores: Auditory Comprehension to assess receptive language skills, Expressive Communication to assess expressive language skills, and Total Language to assess children’s overall language skills. To assess children’s non-verbal intelligence, the Leiter International Performance Scale, 3rd Edition (Leiter-3, Roid et al., 2013) was used. Please refer to Table 1 for group means on each measure.

#### 2.2.2. Novel Word Learning Task

In the novel word learning task, children were asked to learn three different lists of words from three different speakers: a General American-English speaker, a Spanish-accented English speaker, and a Korean-accented English speaker. Each list consisted of four words selected from the Gupta et al. (2004) database. The selected words were based on English phonological rules, the words were bi-syllabic, and followed a CVCVC syllable structure with stress placed on the initial syllable. All of the words across the three lists were matched on biphone probability and neighborhood density (calculated from the online CLEARPOND Database, Marian et al., 2012). Each novel word was paired with a novel object that was selected from the Storkel and Adlof (2009) database. This database provides black and white novel objects normed on semantic set size and neighborhood strength. All of the novel objects selected for the present study were matched on semantic set size, neighborhood strength, and file size as an index of visual complexity. The word-object pairings were counterbalanced across the three conditions. Each novel word learning condition was administered in a separate session, and the order was counterbalanced across children. The novel word learning task was designed and administered using a Tobii T60 XL eye tracker (Tobii Technology AB, Stockholm, Sweden) controlled by E-Prime Professional 2.0.

The set of stimuli was recorded by four female speakers in their mid-30s. Two monolingual English speakers were recruited: one to record the stimuli for teaching the novel words and one to assess the retention of words across the three conditions. Both were native speakers of English with no knowledge of any other languages. The speakers were asked to self-rate the extent of their foreign accent when speaking English on a 10-point scale, with 0 being “none” and 10 being “pervasive” accent. Both speakers self-rated their foreign accent in English as a 0 (none).

Two speakers were recruited to record the stimuli for the accented conditions: a native Spanish speaker who spoke English with a Spanish accent and a native Korean speaker who spoke English with a Korean accent. The Spanish-accented English speaker was from Chiapas, Mexico, and she learned English as an adult. The speaker self-rated her ability to speak English as a 4 (slightly less than adequate) and her foreign accent in English as an 8 (very heavy). The fourth speaker was a native Korean speaker from Seoul, South Korea, who learned English as an adult. The speaker self-rated her ability to speak English as a 4 (slightly less than adequate) and her foreign accent in English as 9 (extremely heavy). In addition to speaker self-ratings, 48 monolingual English-speaking adult listeners (aged 19–47 years) rated the strength of each speaker’s accent. Ratings spanned the same 10-point scale as the self-assessments. Both General American-English speakers were consistently rated as having no foreign accent, whereas the Spanish-accented (*M* = 8.70, *SD* = 1.57) and Korean-accented (*M* = 8.58, *SD* = 1.38) speakers were rated as having heavy foreign accents.

All the stimuli were recorded in a soundproof booth at a 20 kHz sampling rate and normalized to 70 dB amplitude using Praat (version 6.0, Boersma & Weenink, 2015). Please refer to Table 2 for information on each speaker’s fundamental frequency, speech rate, and the average duration of the recordings in each condition.

To further characterize the two accented speakers, each speaker’s productions were phonetically transcribed. The Spanish-accented and Korean-accented speakers differed systematically in their realization of the novel words. The Spanish-accented speaker largely preserved target prosodic structure, consistently maintaining initial stress and full syllable structure across items (e.g., dulek [ˈdulek], tepate [ˈtepeɪt]). Deviations were primarily segmental and reflected systematic mapping to a five-vowel system (e.g., /ɛ/ → [e], /ɪ/ → [i], /ɑ/ → [a]), along with occasional rhotic realization as [ɾ] and minor variation in word-final consonants (e.g., [ˈpedid̚]). In contrast, the Korean-accented speaker exhibited more extensive phonological restructuring. Productions frequently involved a shift of primary stress to the second syllable (e.g., botefe [boʊˈtif], dulek [duˈlɛk]). In addition, word-final consonants were often weakened or unreleased (e.g., [m̚], [f̚], [d̚]), and vowel realizations showed simplification of diphthongs and increased variability. Together, these patterns indicate that while both accented speakers differed from the native speaker, the Spanish-accented speaker preserved key prosodic and structural properties of the target forms to a greater extent than the Korean-accented speaker. Phonetic transcriptions for all stimuli are available in Appendix A.

The novel word learning task consisted of three phases: an introduction, a teaching phase, and a testing phase. Prior to the task, a standard 9-point calibration procedure was conducted using the Tobii eye tracker to ensure accurate measurement of children’s gaze. The introduction consisted of a video of a female cartoon character introducing herself and stating that she will show the child four new toys and teach the child the name of each toy, one by one. Then, children viewed a video of each novel word-object pair, presented in a randomized order for each child. The video showed one novel object on the screen, and children heard a brief description of the object. The novel words were embedded in English phrases. For example, “Look, this toy is called a botefe. A botefe is used to swim. A botefe is so much fun. Look at the botefe”. Semantic context was provided in order to aid learning and maximize the number of exposures to each word. Children heard each novel word four times. The verbs used to describe the function of each toy were selected to have an age-of-acquisition between 3 and 4 years of age.

The testing phase followed immediately after the teaching phase. Children saw a different female cartoon character who spoke with a General American-English accent and introduced the testing phase by saying, “Let’s see how many you remember!” The same test-phase character was used across all three novel word learning conditions. A gaze-contingent stimulus presentation was used in the testing phase. First, children saw two objects for 2000 ms, then a fixation video appeared in the middle. The video played on the screen until the child fixated on the video for 100 ms; otherwise, the video remained on the screen for 10 s. Then, children heard instructions to “Look at the (novel name for one of the objects on the screen).” The objects stayed on the screen for 4000 ms. Each novel word-object pair was tested three times for a total of 12 trials. Each object served equally often as a target and as a distractor, and the location of the object image on the screen was counterbalanced. The order of presentation was pseudorandomized to avoid repetitions of the same target consecutively.

#### 2.2.3. Bilingual Variables

Three bilingual variables were derived from parent reports: length of bilingualism, the number of non-native speakers in the child’s environment, and the strength of accent of individuals in the child’s environment.

#### 2.2.4. Length of Bilingualism

To calculate the length of bilingualism, the time (in years) when the child began learning their second language was subtracted from the child’s chronological age. If the child learned both English and Spanish from birth, the length of bilingualism would be their entire life.

#### 2.2.5. Number of Non-Native English Speakers

Parents were asked to complete a questionnaire specifically designed for the present study, the Summary of Exposure to Accent Questionnaire (SEA-Q). In this questionnaire, parents listed all of the individuals the child interacts with regularly. Then, parents listed all of the languages each individual speaks, the percentage of time they speak each language to the child, the strength of the foreign accent in each language, and how often each person interacts with the child. The total number of non-native English speakers was the sum of all of the non-native English speakers in the child’s environment. The number of non-native English speakers only was considered because the present study was conducted in English, and most of the children did not hear Spanish from non-native speakers.

#### 2.2.6. Strength of Foreign Accent in English

The strength of the foreign accent in English was calculated from the SEA-Q. The strength of a foreign accent in English for each individual was indicated on a 10-point scale with 0 being “none” and 10 being “pervasive.” A weighted strength of accent was calculated for each individual in order to account for how often each individual interacts with the child and how often they speak in English with the child. It is easy to imagine that even though a child may have a caregiver or a relative who speaks English with a foreign accent, it may be the case that this individual does not use English with the child and/or does not interact with the child daily. To account for this variability, the following formula was used: (Proportion of time spent using English when interacting with the child × Strength of accent in English)/(Number of days the person interacts with the child ÷ 7 days of the week). Then, an average weighted strength of accent was calculated for each child.

### 2.3. Analyses

Growth curves were modeled to examine looking patterns to the target image across the three conditions using R (version 3.5; R Core Team, 2018) with the eyetrackingR (Dink & Ferguson, 2015) and the lme4 (Bates et al., 2015) packages. Data were prepared by averaging together the x-y coordinates from both eyes and mapping the averages onto the areas of interest, the target and the foil images. An empirical logit transformation was applied to the number of looks to each image in 50 ms bins (Mirman, 2017). Data cleaning was also conducted in *R*. Only looks to the target and to the foil image were considered, and data cleaning criteria were set to remove any trial with more than 50% missing data; however, there were no trials with 50% or more missing data. The growth curves were calculated for two time windows, an early time window and a late time window (Lany & Saffran, 2010). This was done because a visual inspection of the data revealed possible early and late effects.

Previous literature has traditionally assessed looking patterns for 1800 ms after the onset of the target word (Delle Luche et al., 2015). It takes children about 233 to 367 milliseconds to initiate an eye movement (Ellis et al., 2015). Therefore, the early time window was calculated between 300 ms to 2100 ms. The late time window was calculated from 2100 ms to 3900 ms. The looks over time for the two accented conditions were compared to the looks over time for the General American-English-accented condition, which served as the baseline condition. The models included the empirical logit of looks to the target image as the outcome variable and time and condition as fixed effects. Linear time, quadratic time, and cubic time terms were used in the models. The time terms describe the shape of the growth curves over time, and they reflect how quickly and efficiently children orient to and maintain attention on the target object after hearing the novel word. The linear time variable represents differences in the slope of the time curves, and any significant effect in linear time can be interpreted as the average speed of eye movements across the time period that was analyzed. The quadratic time variable represents differences in steepness or the sharpness of the peak of the growth curves, and any significant effect in the quadratic time can be interpreted as the acceleration of the eye movements across the time period that was analyzed. Similarly, the cubic time variable represents the differences in the steepness or the sharpness of the two peaks of the growth curves, and any significant effect in the cubic time can be interpreted as the change in acceleration and deceleration of eye movements over the time period that was analyzed (Mirman, 2017). The difference between the quadratic and the cubic curves is that the quadratic curve demonstrates the rate of acceleration and deceleration around the central inflection point of the curve, while the cubic term represents the acceleration and deceleration at the tails of the curve during the time window.

Age, socioeconomic status, and non-verbal IQ were considered as covariates, but these variables did not make a contribution to the model, and a model comparison analysis demonstrated no significant difference between the base model with time and condition as fixed effects compared to a model that included age, socioeconomic status, and non-verbal IQ as covariates (*p* > 0.05). Random effects included participant effects for each orthogonal time variable because a model that also included participant-by-condition random effects for each orthogonal time variable did not converge. For the individual difference analyses, three separate models were constructed for each factor. Therefore, each factor was assessed using each condition as a reference group to be able to assess whether each factor was a significant predictor for each condition. Each model included a condition-by-factor interaction to assess whether each factor had a stronger effect for one condition compared to the other conditions. For ease of interpretation, the three conditions were contrast-coded as follows: the General American-English condition was contrast-coded as 0, the Spanish-accented condition was contrast-coded as 1, and the Korean-accented condition was contrast-coded as −1. The models included the orthogonal time variables, the effect of condition, the interaction between condition and the variable of interest, regressed on the empirical logit of looks to image with participants as random effects. The individual-difference models were run on the total duration of the trial (300 ms to 4000 ms).

## 3. Results

### 3.1. Early Time Window

**Figure 1:**
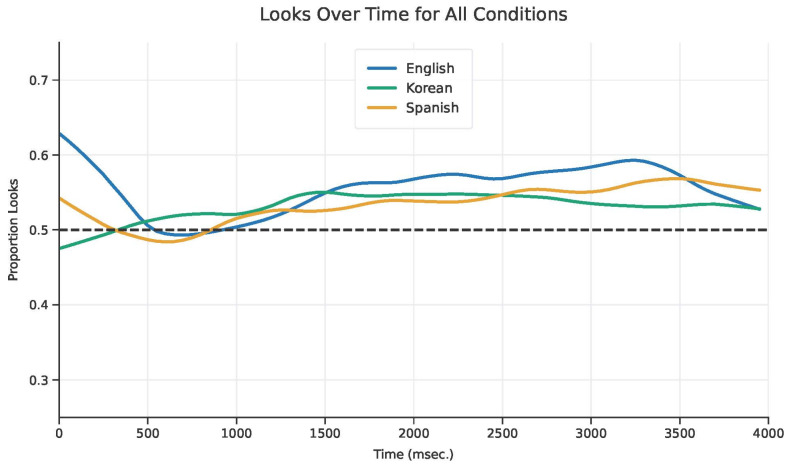
Looks over time for all conditions. The dashed line represents chance performance at 50%.

**Figure 2:**
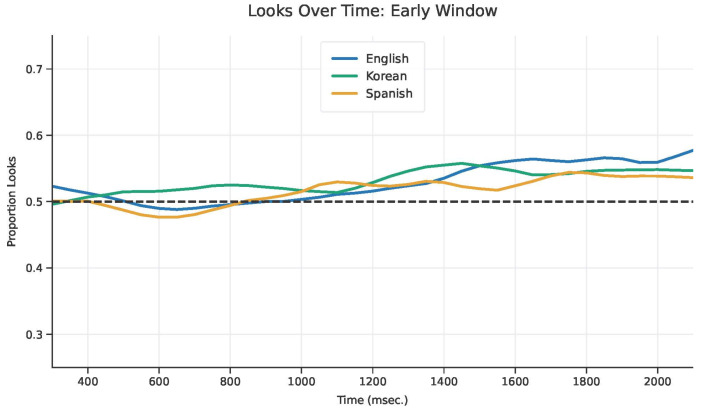
Looks over time for all conditions for the early time window. The dashed line represents chance performance at 50%.

Figure 1 shows children’s looks over time to the target image for the three conditions, and Figure 2 shows children’s looks over time for the early time window. For the early time window between 300 ms and 2100 ms with the General American-English condition as the reference group, the growth curve model revealed a significant effect of condition for the Korean-accented condition (*Estimate* = −0.04, *SE* = 0.02, *p* < 0.05) but not for the Spanish-accented condition (*Estimate* = −0.02, *SE* = 0.02, *p* > 0.05). There was an effect of condition on the linear time term for the Korean-accented condition (*Estimate* = −0.95, *SE* = 0.14, *p* < 0.001) but not for the Spanish-accented condition (*Estimate* = −0.09, *SE* = 0.14, *p* > 0.05). There was an effect of condition on the quadratic time term for the Spanish-accented condition (*Estimate* = 0.42, *SE* = 0.14, *p* < 0.01) but not for the Korean-accented condition (*Estimate* = 0.15, *SE* = 0.14, *p* > 0.05). Condition had a significant effect on the cubic term for the Korean-accented condition (*Estimate* = 0.45, *SE* = 0.14, *p* < 0.01) but not for the Spanish-accented condition (*Estimate* = 0.21, *SE* = 0.14, *p* > 0.05). These findings regarding the time terms indicate that overall, the rate of word learning was higher for the General American-English condition compared to the Korean-accented condition, while the rate of word learning was similar between the General American-English condition and the Spanish-accented condition. The full model results are available in Table 3. Please note that the order in which each condition was administered did not impact children’s performance (*Estimate* = −0.01, *SE* = 0.02, *p* > 0.05).

When the Spanish-accented condition served as the baseline condition, the growth curve model revealed a significant effect of condition, indicating that children had higher fixations to the words in the Korean-accented English condition compared to the Spanish-accented English condition (*Estimate* = −0.07, *SE* = 0.02, *p* < 0.01) in the early time window. There was no effect of condition on the linear time term (*Estimate* = 0.01, *SE* = 0.10, *p* > 0.05). Likewise, there was no effect of condition on the quadratic time term (*Estimate* = −0.07, *SE* = 0.14, *p* > 0.05), nor on the cubic term (*Estimate* = −0.15, *SE* = 0.14, *p* > 0.05). The full model results are available in Table 4.

### 3.2. Late Time Window

**Figure 3:**
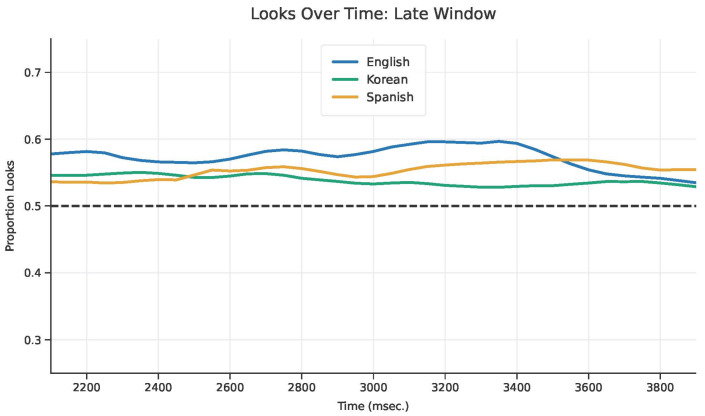
Looks over time for all conditions for the late time window. The dashed line represents chance performance at 50%.

For the late time window between 2100 ms and 3900 ms with the General American-English condition as the reference group, the growth curve model revealed a significant effect of condition for the Korean-accented condition (*Estimate* = −0.15, *SE* = 0.02, *p* < 0.001) but not for the Spanish-accented condition (*Estimate* = −0.04, *SE* = 0.02, *p* > 0.05) indicating that children had higher fixations to the words in the General American-English condition compared to the Korean-accented condition but not for the Spanish-accented condition. There was an effect of condition on the linear time term for the Korean-accented condition (*Estimate* = −0.27, *SE* = 0.14, *p* = 0.05) but not for the Spanish-accented condition (*Estimate* = 0.25, *SE* = 0.14, *p* > 0.05). There was also an effect of condition on the quadratic time term for the Korean-accented condition (*Estimate* = 0.35, *SE* = 0.14, *p* < 0.01) but not for the Spanish-accented condition (*Estimate* = 0.09, *SE* = 0.14, *p* > 0.05). Condition also had a significant effect on the cubic term for the Korean-accented condition (*Estimate* = 0.35, *SE* = 0.13, *p* < 0.01) but not for the Spanish-accented condition (*Estimate* = 0.17, *SE* = 0.13, *p* > 0.05). These findings regarding the time terms indicate that overall, the rate of word learning was higher for the General American-English condition compared to the Korean-accented condition, while the rate of word learning was similar between the General American-English condition and the Spanish-accented condition. The full model results are available in Table 5, and a visual representation of children’s looks over time for the later time window is in Figure 3. Please note that the order in which each condition was administered did not impact children’s performance (*Estimate* = 0.05, *SE* = 0.03, *p* > 0.05).

When the Spanish condition served as the baseline condition, the growth curve model revealed a significant effect of condition, indicating that children had higher fixations to the words in the Spanish-accented condition compared to the Korean-accented condition (*Estimate* = 0.13, *SE* = 0.02, *p* < 0.001). There was an effect of condition on the linear time term (*Estimate* = 0.56, *SE* = 0.13, *p* < 0.001), but not on the quadratic time term (*Estimate* = −0.21, *SE* = 0.13, *p* > 0.05), or on the cubic term (*Estimate* = −0.20, *SE* = 0.13, *p* > 0.05). The full model results are available in Table 6. These findings suggest that children showed a higher rate of word learning in the Spanish-accented condition compared to the Korean-accented condition, unlike in the early time window.

### 3.3. Individual Differences Analyses

#### 3.3.1. Language Skills

When the General American-English condition served as the reference condition, the model revealed a significant main effect of language (*Estimate* = 0.01, *SE* = 0.002, *p* < 0.05), indicating that children with higher language skills outperformed children with lower language skills in the General American-English condition. The model also revealed a significant interaction between language and the Korean-accented condition (*Estimate* = −0.006, SE = 0.001, *p* < 0.001) but not the Spanish-accented condition (*Estimate* = −0.001, *SE* = 0.001, *p* > 0.05), indicating that the effect of language was significantly weaker for the Korean-accented condition compared to the General American-English condition. When the Spanish-accented condition served as the reference condition, the model revealed a significant main effect of language (*Estimate* = 0.01, *SE* = 0.003, *p* < 0.05), indicating that children with higher language skills outperformed children with lower language skills in the Spanish-accented condition. The model also revealed a significant interaction between language and the Korean-accented condition (*Estimate* = −0.006, *SE* = 0.001, *p* < 0.001), indicating that the effect of language was significantly weaker for the Korean-accented condition compared to the Spanish-accented condition. When the Korean-accented condition served as the reference condition, the model revealed a marginally significant main effect of language (*Estimate* = 0.005, *SE* = 0.003, *p* = 0.051). This effect indicates that language skills served as a marginally significant predictor of children’s performance in the Korean-accented condition.

#### 3.3.2. Bilingual Variables

Please refer to Table 7 for correlation analyses between the bilingual variables. The correlations indicate that the variables are weakly and non-significantly related, with only a marginal association between the number of non-native English speakers and the strength of accent (*p* = 0.054).

#### 3.3.3. Length of Bilingualism

On average, children had been bilingual for 3.89 years (*SD* = 1.32, Range: 0.67–5.83 years). When the General American-English condition served as the reference condition, the model did not reveal a significant main effect of children’s length of bilingualism (*Estimate* = 0.04, *SE* = 0.03, *p* > 0.05), indicating that length of bilingualism did not serve as a significant predictor of children’s performance in the General American-English condition. The model did reveal a significant interaction between the Korean-accented condition and length of bilingualism (*Estimate* = 0.03, *SE* = 0.01, *p* < 0.01) but not between the Spanish-accented condition and length of bilingualism (*Estimate* = 0.01, *SE* = 0.01, *p* > 0.05). The significant interaction indicates that the effect of length of bilingualism was stronger for the Korean-accented condition compared to the General American-English condition. When the Spanish-accented condition served as the reference condition, the model did not reveal a significant main effect of children’s length of bilingualism (*Estimate* = 0.05, *SE* = 0.03, *p* > 0.05), indicating that length of bilingualism did not serve as a significant predictor of children’s performance in the Spanish-accented condition. The model did reveal a significant interaction between the Korean-accented condition and length of bilingualism (*Estimate* = 0.02, *SE* = 0.01, *p* < 0.05), indicating that the effect of length of bilingualism was stronger for the Korean-accented condition compared to the Spanish-accented condition. When the Korean-accented condition served as the reference condition, the model revealed a significant main effect of length of bilingualism (*Estimate* = 0.08, *SE* = 0.03, *p* < 0.05), indicating that children who have been bilingual for a longer period of time outperformed children who have been bilingual for a shorter period of time in the Korean-accented condition, when performance is defined as overall amount of looks to the target image.

#### 3.3.4. Number of Non-Native English Speakers

On average, children were exposed to 1.94 non-native speakers (*SD* = 1.36; Range: 0–5). When the General American-English condition served as the reference group, the model did not reveal a main effect of the number of non-native English speakers (*Estimate* = 0.01, *SE* = 0.04, *p* > 0.05), indicating that children with more and children with fewer non-native English speakers in their environment performed similarly in the General American-English condition. The model did reveal a significant interaction for the Korean-accented condition (*Estimate* = −0.05, *SE* = 0.01, *p* < 0.001) and for the Spanish-accented condition (*Estimate* = −0.03, *SE* = 0.01, *p* < 0.05). These significant interactions indicate that the effect of the number of non-native English speakers was significantly weaker in the two accented conditions compared to the General American-English condition. When the Spanish-accented condition served as the reference condition, the model did not reveal a significant main effect (*Estimate* = −0.02, *SE* = 0.03, *p* > 0.05). The model did reveal a significant interaction between the number of non-native English speakers and the Korean-accented condition (*Estimate* = −0.03, *SE* = 0.01, *p* < 0.05), indicating that the effect of the number of non-native English speakers was weaker for the Korean-accented condition compared to the Spanish-accented condition. When the Korean-accented condition served as the reference group, the model did not reveal a significant main effect (*Estimate* = −0.02, *SE* = 0.03, *p* > 0.05), indicating that children with more and children with fewer non-native English speakers performed similarly in the Korean-accented condition.

#### 3.3.5. Strength of Accent

The average weighted strength of accent was 1.31 (*SD* = 2.23, Range: 0–6.17). When the General American-English condition served as the reference condition, the model revealed a significant and negative main effect of strength of accent (*Estimate* = −0.05, *SE* = 0.02, *p* < 0.01), indicating that children exposed to individuals with a slight foreign accent in English outperformed children exposed to individuals with a stronger foreign accent in English. The model also revealed a significant interaction for both the Spanish-accented condition (*Estimate* = 0.06, *SE* = 0.01, *p* < 0.001) and the Korean-accented condition (*Estimate* = 0.03, *SE* = 0.01, *p* < 0.05). These significant interactions indicate that the effect of the strength of accent in English was significantly stronger for the two accented conditions compared to the General American-English condition. When the Spanish-accented condition served as the reference condition, the model did not reveal a significant main effect of strength of accent (*Estimate* = −0.01, *SE* = 0.02, *p* > 0.05). The model did reveal a significant interaction between the Korean-accented condition and the strength of accent (*Estimate* = 0.03, *SE* = 0.01, *p* < 0.001), indicating that the effect of strength of accent was stronger for the Korean-accented condition compared to the Spanish-accented condition. When the Korean-accented condition served as the reference group, the model did not reveal a significant main effect (*Estimate* = 0.01, *SE* = 0.01, *p* > 0.05), indicating that the strength of accent did not serve as a significant predictor of children’s performance in the Korean-accented condition.

## 4. Discussion

The present study examined bilingual children’s ability to learn novel words from a speaker of a familiar accent, Spanish-accented English, and from a speaker of an unfamiliar accent, Korean-accented English, compared to learning from a speaker of General American English. Results revealed that Spanish-English bilingual children performed equally well in the General American-English condition and the Spanish-accented English condition, but performed better in the General American-English condition than the Korean-accented condition. The contrast between the two accented conditions yielded a more complicated result. Spanish-English bilingual children performed better in the Korean-accented than the Spanish-accented condition in the early part of the testing trial. However, this effect was not long-lasting, and in the later part of the testing trial, children’s performance in the Korean-accented condition declined, with children showing better learning in the Spanish-accented condition. These findings suggest that an unfamiliar accent may capture attention earlier in the test trial, but a familiar accent yields a more enduring benefit.

In the present study, bilingual children experienced greater difficulty learning from an unfamiliar accented speaker, the Korean-accented English speaker. Given the robust findings that foreign-accented speech can increase processing (Barker & Turner, 2014; Bent, 2014; Bent & Atagi, 2015; Schmale & Seidl, 2009) and comprehension demands (Holtby, 2010; Mulak & Best, 2013; Nathan et al., 1998; O’Connor & Gibbon, 2011), the present findings for the Korean condition are in line with recent work demonstrating that bilingualism and/or experience with a particular foreign accent does not aid children’s processing of an unfamiliar accent (Evans & Tomé Lourido, 2019; Levy et al., 2019). Importantly, children nonetheless demonstrated successful learning across conditions, suggesting that unfamiliar accented input increases the effort required for learning rather than preventing learning altogether.

At the same time, the present findings align with studies showing better performance when the listener’s and speaker’s language backgrounds match (Bent & Bradlow, 2003; Hanulíková & Weber, 2012; Levy et al., 2019; Pinet et al., 2011). The present study extended these findings to word learning and showed that Spanish-English bilingual children learned equally well in the Spanish-accented English condition and the General American-English condition. Notably, the current findings diverge from recent work on language processing and comprehension. Specifically, McDonald et al. (2018) found that simultaneous Spanish-English bilinguals and early Spanish-English bilinguals experienced processing costs similar to their monolingual peers when processing Spanish-accented English. One reason for the difference in results observed in the present study compared to McDonald et al.’s (2018) study may be due to task design. Specifically, in McDonald et al.’s (2018) study, trial order was randomized, meaning that children could not predict whether they would hear an accented or an unaccented sentence. Conversely, in the present study, the presentation of the trials was blocked by the speaker such that children learned four novel words from each speaker on a separate day. Therefore, the blocked nature of the trial presentation in the present study may have facilitated accommodation to the familiar accent. At face value, it appears that experience with two languages can boost learning, but only in situations where the speaker’s and the listener’s language backgrounds match. However, when bilingual experience factors were assessed, one particular factor was associated with children’s learning from an unfamiliar accented speaker.

### 4.1. Factors Impacting Children’s Learning from Accented Input

The present findings showed that children’s language skills were positively associated with learning from the General American-English speaker and the Spanish-accented speaker. However, language skills were only marginally associated with children’s ability to learn from the Korean-accented speaker. The present study adds to the current literature that has demonstrated positive effects of children’s vocabulary (Banks et al., 2015; Bent, 2014; Bent et al., 2016; Janse & Adank, 2012; McDonald et al., 2018) and more broad language skills (Frizelle et al., 2018) on performance on a variety of tasks involving accented input, but extends these finding to a familiar accent only. It appears that the ability to learn from an unfamiliar accented speaker is associated more with child-external factors, length of bilingualism in particular, than child-internal factors. In the present study, the language assessment tool was used to provide a conceptual score, resulting in one overall score that takes into consideration both children’s English and Spanish language skills. Future work would benefit from assessing each language separately, that is, assessing whether English and Spanish language skills separately are related to accented processing and learning.

### 4.2. The Bilingual Experience

Levy et al.’s (2019) study demonstrated that exposure to two languages did not aid children’s ability to process an unfamiliar accent. It is possible that the crucial aspect of bilingualism that moderates the ability to process accented input is not exposure to two languages, but exposure to non-native and accented speakers of a language. In the current sample, one bilingual factor played a role in children’s ability to learn from accented input. Specifically, the length of time a child had been bilingual played a significant role in the child’s ability to learn from the unfamiliar accented speaker. The longer a child had been bilingual, the better they learned from the Korean-accented speaker. Therefore, longer experience with two languages appears to result in better ability to accommodate to phonological variability during word learning. However, the length of bilingual experience likely encompasses many different factors that may influence children’s word learning performance, including experience with accented input. To zero in on the role of experience with accented input in particular, two other variables were examined to assess whether the number of non-native speakers and the heaviness of their accent were specifically associated with bilingual children’s word learning performance.

The findings were somewhat surprising in that exposure to more non-native speakers of English (i.e., to more native Spanish speakers) did not predict children’s performance in any of the conditions. However, although the number of non-native speakers was not a significant predictor for performance in any of the conditions, the interaction analysis demonstrated that the magnitude of the effect significantly differed among the three conditions. Specifically, exposure to more native speakers was associated with better performance in the General American-English condition and worse performance in the Korean-accented condition. Further, more exposure to speakers with a stronger accent was associated with reduced ability to learn from the General American-English speaker. These patterns suggest that variability in accent exposure may shape how children allocate attention to different types of input, rather than uniformly facilitating or hindering learning. In this, the present findings align with prior studies indicating that the presence of non-native speakers in the child’s environment may influence bilingual children’s language outcomes (Place & Hoff, 2011, 2016). However, the present results indicate that features of accented input, such as cumulative accent strength, may influence the demands associated with learning under specific conditions rather than posing a general obstacle to learning.

It is worth noting that because accent-exposure data was obtained through parent report (the SEA-Q), which was not validated against other measures, and because only one parent was asked to rate the accent of all of the other individuals in the child’s environment, the indexes of exposure may not reliably capture these parameters. Future research should consider gathering language samples and having outside raters rate the speakers’ perceived foreign accent in English. However, this approach poses its own challenges as language samples would most likely be obtained from only one parent, but not the rest of the individuals in the child’s environment. A more complete recording of the child’s environment, such as one using the LENA recording software (Gilkerson & Richards, 2008) may aid in better quantifying children’s exposure to accented input.

An additional consideration in interpreting the present findings is that the General American-English speaker served as the baseline condition and represents a standard and highly familiar variety of English, whereas the Spanish-accented and Korean-accented speakers represent non-native varieties. As such, the observed differences across conditions may reflect not only accent familiarity but also differences associated with standard versus non-standard varieties, including sociolinguistic status and listeners’ expectations about speech. The present design does not allow these factors to be fully disentangled. With respect to unfamiliar but standard varieties of English (e.g., New Zealand English), performance would likely depend primarily on listeners’ prior exposure and familiarity. However, unfamiliar standard accents may impose reduced processing demands relative to non-native accents, given greater overlap in phonological structure and more familiar acoustic-phonetic patterns. Future research should directly compare unfamiliar native varieties and non-native accents to more precisely isolate the contributions of familiarity, accent type, and sociolinguistic expectations.

A related consideration is whether differences across conditions may reflect speaker-specific characteristics rather than accent per se. Several aspects of the design mitigate this possibility. Children did not view the speakers during stimulus presentation, as all auditory stimuli were paired with standardized cartoon characters, thereby controlling visual input across conditions. In addition, all speakers were female and of comparable age, and recordings were produced in the same sound-attenuated environment and normalized for amplitude. Acoustic analyses further indicated no significant differences across speakers in fundamental frequency or speech rate. Together, these factors reduce the likelihood that performance differences were driven by idiosyncratic speaker properties. However, because each accent condition was represented by a single speaker, it is not possible to fully separate accent effects from speaker-specific variation. Future research should include multiple speakers per accent to more precisely isolate the role of accent in word learning. Lastly, a source of variability in the sample relates to children’s language dominance. Parent report indicated that some children were English-dominant, some were Spanish-dominant, and others were more balanced bilinguals. Because the primary focus of the present study was on learning from accented input rather than group differences based on dominance, and because manipulation of accent was within-participants, language dominance was not included as a predictor in the main analyses. However, it is possible that dominance interacts with accent familiarity and processing, and future research should examine how language dominance shapes learning from familiar and unfamiliar accented input.

### 4.3. Conclusions

In conclusion, the present study asked whether the familiar Spanish accent and an unfamiliar Korean accent would impact word learning in Spanish-English bilingual children. Results revealed that the presence of an unfamiliar accent impacted children’s learning in comparison to the two familiar accents. However, there was an association between the length of time a child had been bilingual and the child’s ability to learn novel words from the unfamiliar accented speaker. Therefore, experience with two languages for an extended period of time appears to aid children’s ability to learn from individuals who speak with an unfamiliar accent. The current study also revealed an association between the strength of foreign accent in English and children’s ability to learn from the General American-English speaker, such that greater exposure to stronger accents was linked to reduced performance in this condition. This pattern suggests that variability in accent input may influence the demands associated with learning from different types of speech input.

These results may have implications for understanding how children learn from variable input, but they should be interpreted with caution. For instance, while the results may suggest that characteristics of input, such as accent strength, can shape learning under specific conditions, they do not support broad conclusions about the effects of accented input on language development. Similarly, although these findings may be informative for educational and clinical contexts, they should not be taken as direct guidance for practice. Rather, they highlight the need for further research to examine how accent variability interacts with child-specific factors, such as language skills, across a wider range of settings and populations.

The present study is the first step in deciphering the impact of accented input on children’s ability to learn novel words. Further work is necessary in order to understand all the nuances of accented input. For instance, future studies must consider whether there is a threshold to accented input exposure effects, that is, how much accent variability can be present while still supporting effective language learning? Given the heterogeneity of the bilingual experience, future work is necessary to disentangle the specific variables that may aid learning from an unfamiliar vs. a familiar accent for specific groups of bilinguals, such as simultaneous bilingual children compared to sequential bilingual children. Although these and many more questions remain to be answered, the present study contributes novel findings regarding children’s flexibility to accommodate to variability in their input during language learning.

## Figures and Tables

**Table 1:** Participant characteristics.

	Group Performance *Mean* (*SD*)
*n*	46
Gender	17 boys, 29 girls
Age (years)	4.88 (0.60)
Socioeconomic Status	14.42 (4.83)
English Age of Acquisition (months)	9.74 (13.22)
Spanish Age of Acquisition (months)	1.22 (7.10)
English Daily Exposure (%)	44.06 (16.63)
Spanish Daily Exposure (%)	55.94 (16.56)
Non-verbal IQ	103.22 (7.11)
Receptive Language	112.59 (13.89)
Expressive Language	108.54 (14.08)
Total Language	111.52 (14.66)

Note. Language skills were assessed using the Preschool Language Scales-5th Edition, Spanish Edition. Non-verbal intelligence was assessed using the Leiter-3. Language and non-verbal intelligence scores are standard scores.

**Table 2:** Speaker characteristics and duration of speech in each condition.

	General American-English Speaker	Spanish-Accented English Speaker	Korean-Accented English Speaker
Fundamental Frequency (Hz)	217.27 (10.93)	221.15 (6.98)	202.18 (5.96)
Speech Rate (syllable/second)	2.73 (0.16)	2.33 (0.17)	2.36 (0.15)
Average Duration of Speech (seconds)	10.67 (1.13)	13.34 (0.60)	10.56 (0.47)

Note. Speech rate was calculated by dividing the total number of syllables spoken by the total time (in seconds); thus, speech rate is expressed in syllables per second. Non-parametric Kruskal–Wallis tests did not reveal any significant differences in the speakers’ acoustic characteristics. Speech rate was calculated based on each speaker’s reading of the *Grandfather Passage* (Darley et al., 1975).

**Table 3:** Parameter Estimates for Comparing Performance Across the Three Conditions for the Early Time Window.

	Estimate	Standard Error	*t*-Value
Intercept	0.15	0.04	3.49 ***
Linear	0.88	0.24	3.69 ***
Quadratic	−0.56	0.19	−2.92 **
Cubic	−0.14	0.16	−0.86
Korean Condition	−0.04	0.02	−2.57 **
Spanish Condition	−0.02	0.02	−1.46
Linear: Korean Condition	−0.95	0.14	−6.77 ***
Linear: Spanish Condition	−0.09	0.14	−0.68
Quadratic: Korean Condition	0.15	0.14	1.08
Quadratic: Spanish Condition	0.42	0.14	2.99 **
Cubic: Korean Condition	0.45	0.14	3.19 **
Cubic: Spanish Condition	0.21	0.14	1.47

*** *p* < 0.001; ** *p* < 0.01; * *p* < 0.05.

**Table 4:** Parameter Estimates for the Spanish-accented Condition vs. the Korean-accented Condition for the Early Time Window.

	Estimate	Standard Error	*t*-Value
Intercept	0.11	0.04	2.78 **
Linear	0.42	0.24	1.73
Quadratic	−0.07	0.14	−0.51
Cubic	−0.11	0.09	−1.17
Korean Condition	−0.07	0.02	−3.25 **
Linear: Korean Condition	0.01	0.1	0.10
Quadratic: Korean Condition	−0.07	0.14	−0.47
Cubic: Korean Condition	−0.15	0.14	−1.10

*** *p* < 0.001; ** *p* < 0.01; * *p* < 0.05.

**Table 5:** Parameter Estimates for Comparing Performance Across the Three Conditions for the Late Time Window.

	Estimate	Standard Error	*t*-Value
Intercept	0.24	0.06	3.92 ***
Linear	0.09	0.15	0.62
Quadratic	−0.18	0.13	−1.45
Cubic	−0.28	0.10	−2.70 **
Korean Condition	−0.15	0.02	−6.54 ***
Spanish Condition	−0.04	0.02	−1.96 *
Linear: Korean Condition	−0.27	0.14	−1.94 *
Linear: Spanish Condition	0.25	0.14	1.82
Quadratic: Korean Condition	0.35	0.14	2.57 **
Quadratic: Spanish Condition	0.09	0.14	0.67
Cubic: Korean Condition	0.35	0.13	2.61 **
Cubic: Spanish Condition	0.17	0.13	1.26

*** *p* < 0.001; ** *p* < 0.01; * *p* < 0.05.

**Table 6:** Parameter Estimates for the Spanish-accented Condition vs. the Korean-accented Condition for the Late Time Window.

	Estimate	Standard Error	*t*-Value
Intercept	0.14	0.07	2.05
Linear	0.15	0.17	0.88
Quadratic	0.04	0.12	0.36
Cubic	−0.04	0.08	−0.52
Korean Condition	0.13	0.02	5.60 ***
Linear: Korean Condition	0.56	0.13	4.17 ***
Quadratic: Korean Condition	−0.21	0.13	−1.53
Cubic: Korean Condition	−0.20	0.13	−1.52

*** *p* < 0.001; ** *p* < 0.01; * *p* < 0.05.

**Table 7:** Pearson Correlations for Bilingual Variables.

	Length of Bilingualism	Number Non-Native English Speakers	Strength of Accent
Length of Bilingualism	--	−0.13	0.07
Number Non-native English Speakers	−0.13	--	0.27
Strength of Accent	0.07	0.27	--

## Data Availability

The raw data supporting the conclusions of this article will be made available by the authors on reasonable request. The data presented in this study are not publicly available at this time because they form part of an ongoing research program and are being analyzed for additional planned publications addressing separate research questions.

## Note

1 We use the term “accented speech” as a shorthand to refer to speech produced in varieties that differ from the local norm (e.g., General American English). Our use of this term reflects relative differences in accent familiarity rather than the presence versus absence of an accent.

## Appendix A

**Table A1:** Phonemic targets and phonetic realizations of novel words across speakers.

Word	Target	General American English Speaker	Spanish Speaker	Korean Speaker
bafere	/ˈbæfɪr/	[ˈbæfɪɹ]	[ˈbaːfiɾ]	[baˈfɪɹ]
botefe	/ˈbɑtif/	[ˈbɑtif]	[ˈbatef]	[boʊˈtif]
dinume	/ˈdaɪnum/	[ˈdaɪnum]	[ˈdainum]	[ˈdɪnum̚]
dulek	/ˈdulɛk/	[ˈdulɛk]	[ˈdulek]	[duˈlɛk]
getov	/ˈgɛtɑv/	[ˈgɛtɑv]	[ˈgetav]	[gɛˈtɑv]
gonepe	/ˈgoʊnip/	[ˈgoʊnip]	[ˈgonip]	[goʊˈnif̚]
kitoge	/ˈkɪtoʊg/	[ˈkɪtoʊg]	[ˈkigoʊh]	[kɪˈtoʊg̚]
kosof	/ˈkɑsɑf/	[ˈkɑsɑf]	[ˈkasaf]	[kɑˈsɑf]
pedede	/ˈpɛdid/	[ˈpɛdid]	[ˈpedid̚]	[pɛˈdɪd̚]
pomive	/ˈpoʊmaɪv/	[ˈpoʊmaɪv]	[ˈpomaɪv]	[poʊˈmɪm]
tepate	/ˈtɛpeɪt/	[ˈtɛpeɪt]	[ˈtepeɪt]	[tɛˈpeɪt̚]
tinade	/ˈtɪneɪd/	[ˈtɪneɪd]	[ˈtineɪd]	[tɪˈneɪd̚]

Note. Novel words were obtained from Gupta et al. (2004). Both General American-English speakers produced the stimuli with target-like phonological structure; therefore, only one set of productions is shown above.
